# Evaluating the efficacy of microbial antagonists in inducing resistance, promoting growth, and providing biological control against powdery mildew in wheat

**DOI:** 10.3389/fmicb.2024.1419547

**Published:** 2024-07-23

**Authors:** Kariyappa R. Choudaker, Vaibhav Kumar Singh, Abhijeet Shankar Kashyap, Aakash V. Patel, Koshal K. Sameriya, Dhananjay Yadav, Nazia Manzar, Deeba Kamil, Lakshman Prasad, M. S. Saharan

**Affiliations:** ^1^Wheat Pathology Laboratory, Division of Plant Pathology, ICAR-Indian Agricultural Research Institute, New Delhi, India; ^2^Molecular Biology Lab, ICAR-National Bureau of Agriculturally Important Microorganisms, Mau, Uttar Pradesh, India

**Keywords:** wheat, microbial antagonist, biocontrol, induce resistance, growth promotion, polyphenol oxidase (PPO), *Pseudomonas fluorescens*, *Bacillus amyloliquefaciens*

## Abstract

This study evaluates the biocontrol efficacy of three bacterial strains *(Pseudomonas fluorescens* DTPF-3, *Bacillus amyloliquefaciens* DTBA-11, and *Bacillus subtilis* DTBS-5) and two fungal strains (*Trichoderma harzianum* Pusa-5SD and *Aspergillus niger* An-27) antagonists, along with their combinations at varying doses (5.0, 7.5, and 10.0 g/kg of seeds), against wheat powdery mildew. The most effective dose (10 g/kg seeds) was further analyzed for its impact on induced resistance and plant growth promotion under greenhouse conditions. The study measured defense enzyme activities, biochemical changes, and post-infection plant growth metrics. All tested microbial antagonists at 10 g/kg significantly reduced PM severity, with *B. subtilis* strain DTBS-5 outperforming others in reducing PM severity and achieving the highest biocontrol efficacy. It was followed by *B. amyloliquefaciens* strain DTBA-11 and *P. fluorescens* strain DTPF-3, with the fungal antagonists showing no significant effect. Wheat crops treated with *B. subtilis* strain DTBS-5 exhibited substantial increases in defense-related enzyme activities and biochemicals, suggesting an induced resistance mechanism. The study found a 45% increase in peroxidase (POD) activity, a 50% increase in catalase (CAT) activity, a 30% increase in phenolic content, and a 25% increase in soluble protein content in the wheat plants treated with microbial antagonists. The study highlights the effectiveness of microbial antagonists, particularly *B. subtilis* strain DTBS-5, in managing wheat PM through biocontrol, induced resistance, and enhanced plant growth, offering a sustainable alternative to chemical treatments.

## Graphical Abstract

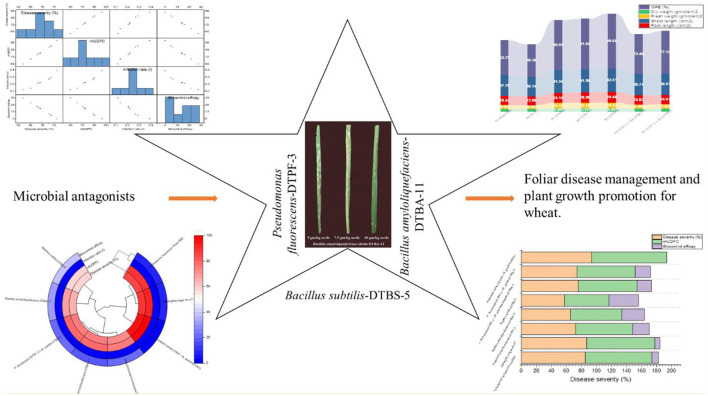

## Introduction

Wheat (*Triticum* species) is considered a major food source for millions of people worldwide. The increasing global population necessitates additional food production, especially in India, which urgently needs to transform its strategies to boost wheat production and productivity. The sustainable productivity of wheat is of utmost importance due to numerous biotic constraints that affect grain quality and limit production and profitability. Powdery mildew (PM), caused by *Blumeria graminis* f.sp. *tritici* (), is a very serious disease that follows the three rusts in importance and can limit yield and cause significant economic losses in wheat globally (up to 25%) (Mwale et al., 2014; Basandrai and Basandrai, 2017). Common methods for reducing PM disease include the development of host resistance and the use of fungicides. However, because pathogen virulence and host resistance evolve simultaneously, qualitative resistance quickly loses its efficacy (McDonald and Linde, 2002). Additionally, there are reports of the emergence of fungicide-resistant strains in the pathogen population (Xia et al., 2002; Yang et al., 2011). The widespread and injudicious use of fungicides can also lead to environmental contamination and health risks. Thus, the need to search for non-chemical alternative methods that provide eco-friendly and economical control of wheat PM has become important. As a cost-efficient and environmentally acceptable solution for PM management, biological control agents (BCAs) reduce the likelihood of fungicide-resistant strains emerging. BCAs are becoming increasingly popular as a successful alternative control method. They provide an additional strategy that may be included in integrated management programs for wheat PM disease to increase protection while maintaining profitability and production. To date, numerous microbial antagonists have been reported to be effective in biocontrolling several PM diseases (Elad et al., 1996; Kiss, 2003; Romero et al., 2004), but very few are currently available on the market as bioformulations/commercial products in several countries (Shishkoff and McGrath, 2002; Punja and Utkhede, 2003). However, the majority of the studies and uses of BCAs have largely been focused on the PM disease of greenhouse vegetables and ornamental crops (Paulitz and Belanger, 2001; Giotis et al., 2012; Medeiros et al., 2012), with very little information available on the uses of microbial antagonists in the biocontrol of wheat PM pathogens (Gao et al., 2015; Russ et al., 2021). Therefore, studies on the evaluation and identification of effective microbial antagonists for bioprotecting wheat against PM disease have become vital. The current study aims to assess the efficacy of several microbial antagonists in bioprotecting plants by inducing systemic resistance and promoting plant growth against *B. graminis tritici* (*Bgt*).

## Materials and methods

### Microbial antagonist collection source

This study used five talcum-based bioformulations of different microbial antagonists (two fungal and three bacterial origins) as bioinoculants. The fungal strains *Trichoderma harzianum* Pusa-5SD (TH-Pusa-5SD) (MTCC No.5371) and *Aspergillus niger* An-27 (AN-An-27) were obtained from the Pulse Pathology Laboratory and three bacterial strains *Pseudomonas fluorescens* DTPF-3 (PF-DTPF-3) (KP017263), *Bacillus amyloliquefaciens* DTBA-11 (BA-DTBA-11) (KF850150), and *Bacillus subtilis* DTBS-5 (BS-DTBS-5) (JQ688022) were obtained from the Plant Bacteriology Laboratory of the Division of Plant Pathology, ICAR-IARI, New Delhi.

### Phytopathogen cultures

The pathogenic culture of *Blumeria graminis* f. sp. *tritici* (*Bgt*) was used as an inoculum. These cultures were a mixture of highly virulent and predominant *Bgt* cultures. The mixed conidial inoculum of *Bgt* cultures was obtained from the Rice and Wheat Research Centre, C.S.K. Himachal Pradesh Agricultural University, Malan, Himachal Pradesh, India. The mixed conidial inoculum of *Bgt* cultures was maintained and increased on the highly susceptible wheat cultivar Agra Local in a temperature-controlled growth chamber, following standard protocols and procedures (Pathania et al., 1996).

### Plant material

The pure seeds of the wheat cultivar PBW 343 were obtained from the Punjab Agricultural University's Department of Plant Breeding and Genetics in Ludhiana, Punjab, India.

### Biological control efficacy of microbials against *B. graminis tritici*

The selected talcum-based bioformulation of all five microbial antagonists and their consortia at three different doses, 5.0 g, 7.5 g, and 10.0 g/kg of seeds, were preliminarily screened for their potential in bioprotecting wheat against PM disease under temperature-controlled greenhouse conditions at the Division of Plant Pathology, ICAR-IARI, New Delhi, India.

### Growing seedlings, seed treatment, and pathogen inoculation

#### Seed sterilization

Wheat seeds were surface sterilized by immersing them in 0.1% sodium hypochlorite for 2 min. This was followed by three rinses with sterile distilled water to remove any residual sterilant. The seeds were then air-dried.

#### Soil preparation

Field soil was collected from the experimental farm of the Department of Plant Pathology, ICAR-IARI, New Delhi, India. The collected soil was used as the potting medium for this study. To ensure sterility, the soil was autoclaved twice at 24-h intervals at 121°C and 15 psi for 20 min each time.

#### Seed coating

The dry, surface-sterilized seeds were coated with 0.2% carboxymethyl cellulose (CMC) to act as an adhesive before sowing. Following the coating, the seeds were treated with talcum-based bioformulation of microbial antagonists at a concentration of 10^8^ cfu/g. The bioformulation was applied at three different doses: 5.0 g, 7.5 g, and 10.0 g per kg of seeds. For the coating process, the seeds were placed in a container, and the bioformulation was sprinkled on them. The seeds were then gently shaken to ensure a uniform coating. The microbial-coated seeds were subsequently scattered on a Petri dish and air-dried overnight at 24 ± 2°C.

#### Provax coating

The seeds treated with Provax-200 were prepared by mixing the fungicide at a concentration of 0.3% (w/w) with the seeds. The seeds were placed in a container, Provax-200 was added, and the mixture was gently shaken to ensure an even coating.

#### Sowing and growing conditions

The treated seeds were sown in greenhouse pots (15 cm in diameter) filled with the sterilized soil. Four pots, each containing 10 seeds, were prepared for each microbial antagonist treatment and their consortia and for the control treatments. The experimental design was a randomized complete block design (RCBD), and the experiment was conducted twice. The pots were maintained in a temperature-controlled greenhouse at 22 ± 2°C, 50–70% relative humidity (RH), and a 12-h daylight cycle. The plants were irrigated on alternate days.

#### Pathogen inoculation

At the tillering and boot leaf stages, the seedlings from each treatment and all replications were challenge-inoculated with a conidial suspension of a mixture of *Blumeria graminis* f. sp. *tritici* (Bgt) cultures. The inoculation was performed using a hand sprayer, following the standard procedure described in Paul et al. (1999).

#### Control treatments

The seeds treated with sterile distilled water only served as the negative control (inoculated with Bgt) and the positive control (untreated and uninoculated healthy plants). The seeds treated with Provax-200 at a concentration of 0.3% (w/w) were used as a fungicide-treated check.

#### Disease scoring and quantification

The disease developments on each plant in all the replications were scored from 14 days post-inoculation (dpi) with *B. graminis tritici*, following the 0 to 9 scale described by Saari and Prescott (1975). Observations were taken at four time points: 14 dpi, 21 dpi, 28 dpi, and 35 dpi. Disease severity (%) was calculated using the following formula:


$$\text{Disease severity }(\%)\;=\frac{\text{ Proportion of leaf area infected }}{\text{Total leaf area assessed}}\;\times 100$$

The area under the disease progress curve (AUDPC) for powdery mildew disease development on each treatment was calculated using the following formula (Milus and Line, 1986):


$$\begin{array}{c} AUDPC=\frac{\left({\text{X}}_{1}+{\text{X}}_{2}\right)}{2}\times \left({\text{t}}_{2}-{\text{t}}_{1}\right)+\frac{\left({\text{X}}_{2}+{\text{X}}_{3}\right)}{2}\times \left({\text{t}}_{3}-{\text{t}}_{2}\right)\\ +\frac{\left({\text{X}}_{3}+{\text{X}}_{4}\right)}{2}\times \left({\text{t}}_{4}-{\text{t}}_{3}\right) \end{array}$$

where X_1_, X_2_, X_3_, and X_4_ refer to the disease severity recorded at 14, 21, 28, and 35 dpi, respectively.

N_1_ is the interval day between X_1_ and X_2_, and N_2_ is the interval day between X_3_ and X_4_.

The relative area under the disease progress curve (rAUDPC) was calculated using the following formula:


$$\begin{array}{l} \text{rAUDPC }=\frac{\text{ line}/\text{genotype AUDPC }}{\text{susceptible AUDPC}}\;\times \;100. \end{array}$$

The apparent infection rate (*r*) was estimated using the following formula (Van der Plank, 1963):


$$\begin{array}{l} r\;=\;\frac{1}{t2\;-\;t1}\;\left(\log_{e}\frac{\;X2}{1-X2}\;-\;log_{e}\frac{\;X1}{1-X1}\;\right), \end{array}$$

where X_1_ is the disease severity recorded at date t_1_, X_2_ is the disease severity recorded at date t_2_, and t_2_ - t_1_ is the interval in days between these dates.

#### Biological control efficacy

The biocontrol efficacy of microbial antagonists was calculated using the following formula (Guo et al., 2004):


$$\begin{array}{l} BCE\;=\frac{\;D_{C}\;-D_{T}}{D_{C}}\;\times \;100, \end{array}$$

where D_C_ is the disease severity (%) of the infected control, recorded at 14, 21, and 28 days post-inoculation (dpi).

D_T_ is the disease severity (%) of the treatment group, recorded at 14, 21, and 28 days post-inoculation (dpi).

### Determination of microbial antagonists for induced resistance abilities

The dose of microbial treatments that showed maximum biocontrol efficiency against powdery mildew disease during preliminary tests was further studied for their induced resistance abilities in wheat cv. PBW 343 upon infection with *B. graminis tritici*. The assessment of various defense-related enzymes, including peroxidase (POD), polyphenol oxidase (PPO), catalase (CAT), and chitinase, along with biochemical tests and total phenol content, was conducted on the wheat seeds treated with microbial antagonists (using seed soaking treatment). These assessments were performed upon infection with *B. graminis tritici* using the most effective dose of the microbial antagonists and their consortia (10 g per kg of seeds). The experiments were carried out under potted-plant conditions in a temperature-controlled greenhouse at the Division of Plant Pathology, ICAR-IARI, New Delhi. Leaves (1 g each) from each treatment in all the replications were taken at different time points, 0, 24, 48, 72, 96, and 120 h post-inoculation (hpi) with *B. graminis tritici*. After homogenizing the leaf samples with 10 ml of phosphate buffer (pH 6.8, 0.1 M), the samples were split into two equal pieces, each holding 5 ml. One part was centrifuged for 15 min at 4°C at 12,000 rpm, and the clear supernatant was used as a source of enzyme. The other 5-ml portion was taken for biochemical activity estimation and analysis.

#### Estimation of peroxidase activity

Peroxidase (POD) activity was quantified using Kashyap et al. (2021)'s protocols. The enzymatic source material was obtained from the assay's supernatant. The composition of the reaction mixture included 1.5 mL of 0.05 M pyrogallol, 0.5 mL of extracted enzyme solution, and 0.5 mL of 1% H_2_O_2_, which was subjected to incubation at a controlled temperature setting of 28°C, allowing a fluctuation range of ±2°C. Initial optical density readings of the reaction mixture were standardized to a baseline of zero at 420 nm wavelength using a spectrophotometric device, with subsequent measurements of optical density alterations occurring at 20-s intervals for a duration of 3 min. A heat-inactivated enzyme preparation served as the negative control within this experimental framework. POD activity levels were deduced from the rate of optical density variation within the reaction mixture, denominated as the alteration in optical density per minute per gram of protein present in the fresh tissue sample.

#### Estimation of polyphenol oxidase activity

Polyphenol oxidase (PPO) activity was quantified following the methodology of Mayer et al. (1965). The assay's reaction mixture comprised 1.5 mL of 0.1 M sodium phosphate buffer at a pH of 6.5 and 200 μL of enzyme extract. The initiation of the enzymatic reaction was marked by the addition of 200 μL of 0.01 M catechol. The reaction mixture was incubated at ambient temperature, and the absorbance was measured at a wavelength of 495 nm to determine PPO activity. Absorbance variations were systematically recorded at 30-s intervals over a span of 2 min. The measure of PPO activity was articulated as the rate of absorbance change per minute per gram of protein in fresh tissue samples.

#### Estimation of catalase activity

Catalase (CAT) activity assessment was performed following Chandlee and Scandalios (1984)'s protocols, incorporating minor adjustments. The supernatant served as the source for the enzyme assay. The enzymatic breakdown of H_2_O_2_ was monitored by reducing the absorbance at 240 nm. The activity of the enzyme was quantified as the rate of decrease in absorbance per minute per gram of protein in fresh tissue samples.

#### Estimation of chitinase activity

Chitinase activity was assessed colorimetrically by quantifying the N-acetyl-D-glucosamine (NAG) liberated from colloidal chitin. The preparation of colloidal chitin was executed in adherence to the methodology of Berger and Reynolds (1958). Chitinase activity was determined by comparing the measured values to a standard curve of NAG.

#### Estimation of phenol content

Total phenolic content was quantified using the Folin-Ciocalteau reagent, as described by Singleton et al. (1999) and Lee et al. (2004). Samples weighing 2 g each were homogenized in 80% ethanol at ambient temperature (25°C), followed by centrifugation at 10,000 rpm for 15 min at a low temperature (4°C), with the supernatant retained. The residue underwent two additional extractions with 80% ethanol, and the solvents were evaporated using a rotary evaporator at 40°C for 1 h. The dried residue was redissolved in 5 mL of distilled water. A 100-μL aliquot of this solution was diluted to 3 mL with water, and 0.5 mL of Folin-Ciocalteau reagent was added. Following a 3-min interval, 2 mL of 20% sodium carbonate solution was introduced, and the mixture was thoroughly mixed. The development of color occurred, and the absorbance was measured at 650 nm using a Bausch and Lomb spectronic-21 UVD spectrometer 60 min post-mixture, with catechol serving as the reference standard. The results were presented as mg catechol/100 g of fresh weight material.

#### Estimation of soluble protein content

The soluble protein content of the enzyme extracts was calculated using the bovine serum albumin Bradford methodology (Bradford, 1976).

### Effect of microbial antagonists on plant growth promoting traits

The pot experiment with the most effective dose of microbial antagonists and their consortia (10 gm/kg seeds) was also conducted to assess plant growth promotion abilities in wheat cv. PBW 343 under greenhouse conditions. This study includes the inoculum concentration of the talc-based bioformulations (10^8^ cfu/gm) and the Bgt (10^5^ conidia/mL). Thirty-five days post-seed treatment (dpt) with microbial antagonists alone and 35 days post-inoculation (dpi) with the *B. graminis tritici* pathogen, whole plants along with roots were uprooted from each treatment in all the replications. The root and shoot of each plant cut from the crown region were measured for plant length (cm). The fresh weight of both root and shoot parts was determined through direct weighing, followed by oven-drying at 60°C for 72 h, after which the dry weight was recorded. Plant length and dry weight parameters were utilized to assess the growth-promoting effects of bacterial antagonists. Evaluations were carried out for the application of microbial antagonists alone and in conjunction with *B. graminis tritici* to determine the relative impact of these antagonists on plant biomass. Comparisons were made between an infected control (plants inoculated with a conidial suspension of *B. graminis tritici*) and a non-treated control (healthy plants without treatment). The calculation of the relative growth promotion efficacy (GPE) attributed to the microbial antagonists was based on the dry weight of the plant (incorporating both root and shoot dry weights), following the methodology described in Singh et al. (2012); Manzar et al. (2021); Mahawer et al. (2023).

### Statistical analysis

The experiments were conducted using a randomized complete block design (RCBD). The presented data are from the illustrative experiments conducted twice. Statistical analysis was conducted, and means were calculated using the Duncan Multiple Range Test (DMRT) to identify significant differences between treatments at the level of *p* of ≤ 0.05. SPSS software (version 16.0) was employed for data analysis.

## Results

### Biocontrol potential of microbial antagonists against *B. graminis tritici*

The objective of the preliminary potted-plant experiments was to ascertain the effects on disease development, rAUDPC, apparent infection rate (r), and biological control efficacy (BCE) in wheat against the powdery mildew pathogen, *B. graminis tritici*, under temperature-controlled greenhouse conditions. All five talcum-based bioformulations of microbials and their consortia were tested at three different doses (5.0 g, 7.5 g, and 10.0 g per kg of seeds).

### Disease severity

**Figure 1:**
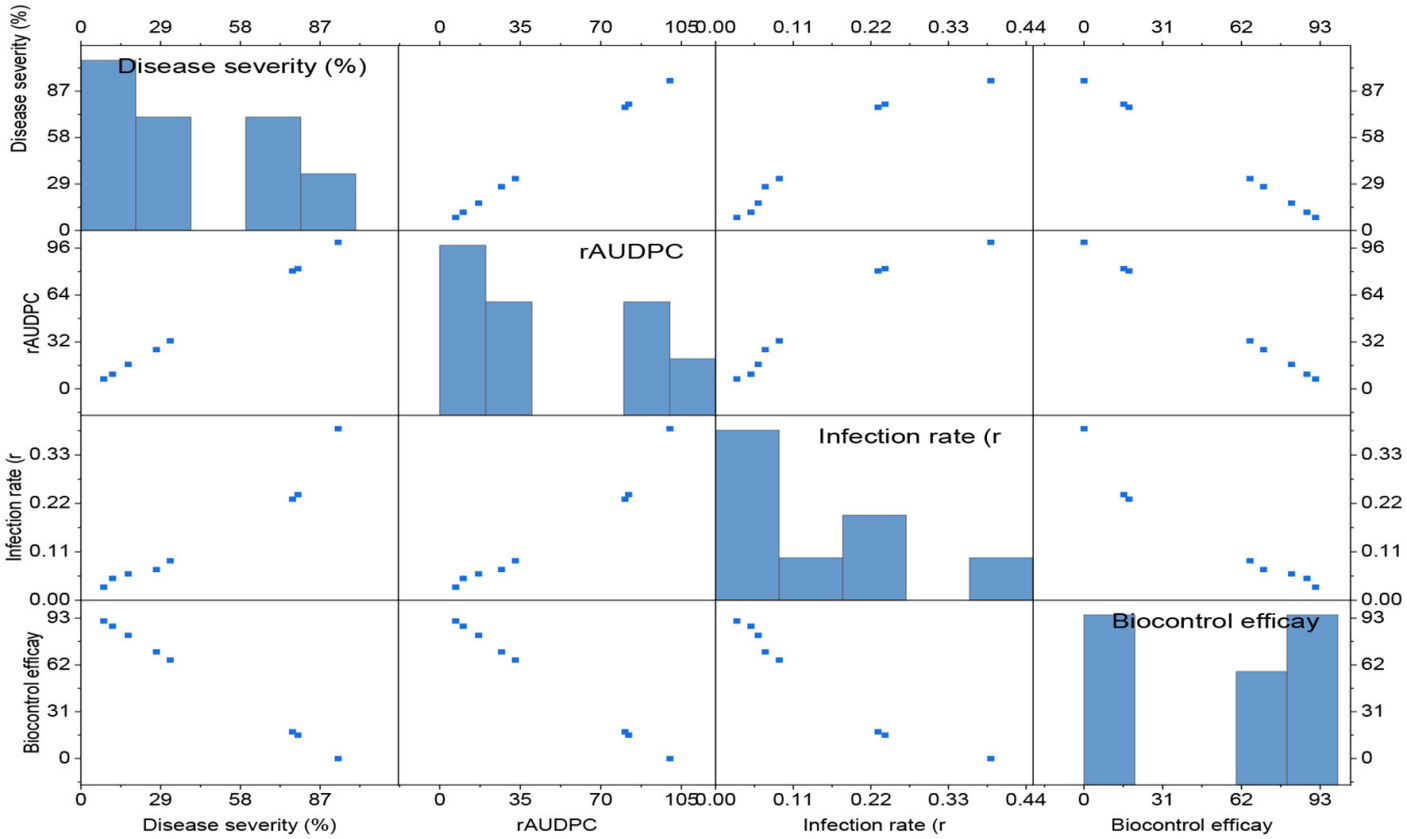
The comparative efficacy of biological control agents (treatment dose of 10 g/kg seeds) against powdery mildew disease parameters. Disease severity (%), rAUDPC, and infection rate (*r*). The data are based on disease observations made four times weekly and a mean of four replicates with 10 plants.

The severity of powdery mildew disease was assessed at weekly intervals, from 14 days post inoculation (dpi) to 35 dpi, across various treatments and replications. The inoculum concentration of the talc-based bioformulations was 10^8^ cfu/gm, and the Bgt was 10^5^ conidia/mL. The results indicated that microbial antagonists and their consortia, applied at a dose of 10 g/kg seeds, significantly reduced disease severity compared to other doses [5.0 g (Supplementary Figure 1) and 7.5 g (Supplementary Figure 2) per kg of seeds]. The infected control exhibited substantially higher mean disease severity (93.56%) than the treated groups. The seeds treated with *Bacillus subtilis* DTBS-5 (BS-DTBS-5) at 10 g/kg demonstrated superior efficacy, with the lowest mean disease severity (8.24%) and maximum biocontrol efficacy (91.19%). BA-DTBA-11 and PF-DTPF-3 were also used at the same dose, exhibiting mean disease severities of 11.44% and 17.14% and mean biocontrol efficacies of 87.77% and 81.68%, respectively. Consortia treatments *PF-*DTPF-3 + *BS*-DTBS-5 and *PF-*DTPF-3 + *BA-*DTBA-11 at 10 g/kg seeds were moderately effective, with mean disease severities of 27.41% and 32.45% and mean biocontrol efficacies of 70.70% and 65.31%, respectively. However, the fungal antagonists *TH-*Pusa-5SD and *AN-*An-27 at each dose showed limited efficacy against powdery mildew. The scattered plot matrix appears to have a strong negative correlation between disease severity and biocontrol efficacy, as higher efficacy leads to lower disease severity (Figure 1). *BS-*DTBS-5, *BA-*DTBA-11, and *PF-*DTPF-3 at 10 g/kg seeds exhibited superior biocontrol efficacy, making them promising candidates for controlling powdery mildew in wheat.

### Progression of disease

**Figure 2:**
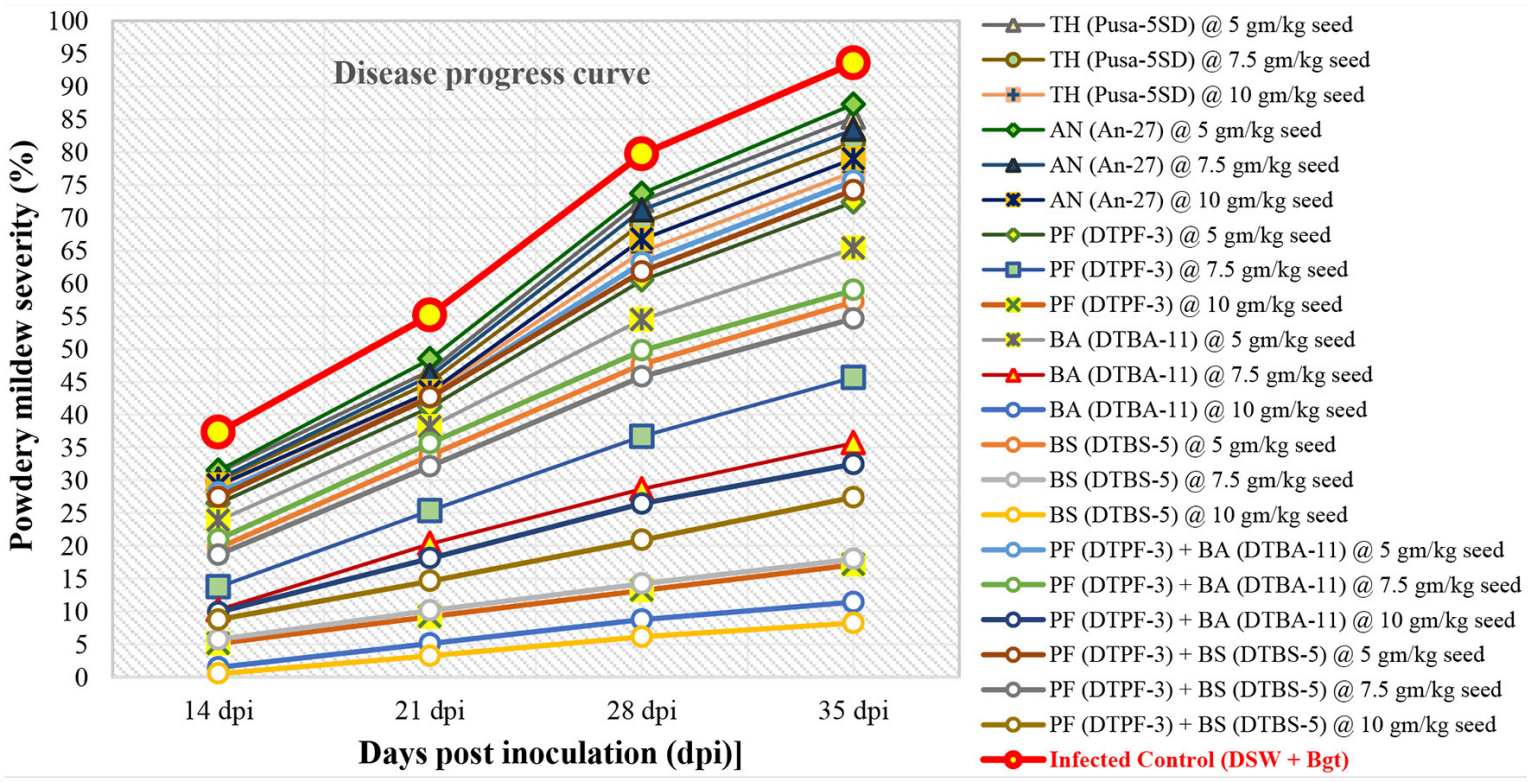
Development of powdery mildew caused by *B. graminis tritici* on wheat cv. PBW 343 treated with microbials and their consortia under greenhouse conditions [TH (Pusa-5SD): *Trichoderma harzianum* strain Pusa-5SD; AN (An-27): *Aspergillus niger* strain An-27; PF (DTPF-3): *Pseudomonas fluorescens* strain DTPF-3; BA (DTBA-11): *Bacillus amyloliquefaciens* strain DTBA-11; and BS (DTBS-5): *Bacillus subtilis* strain DTBS-5]. DSW: sterile distilled water; *Bgt*: *B. graminis* f. sp. *tritici*. The data are based on disease observations made four times weekly and a mean of four replicates with 10 plants.

**Figure 3:**
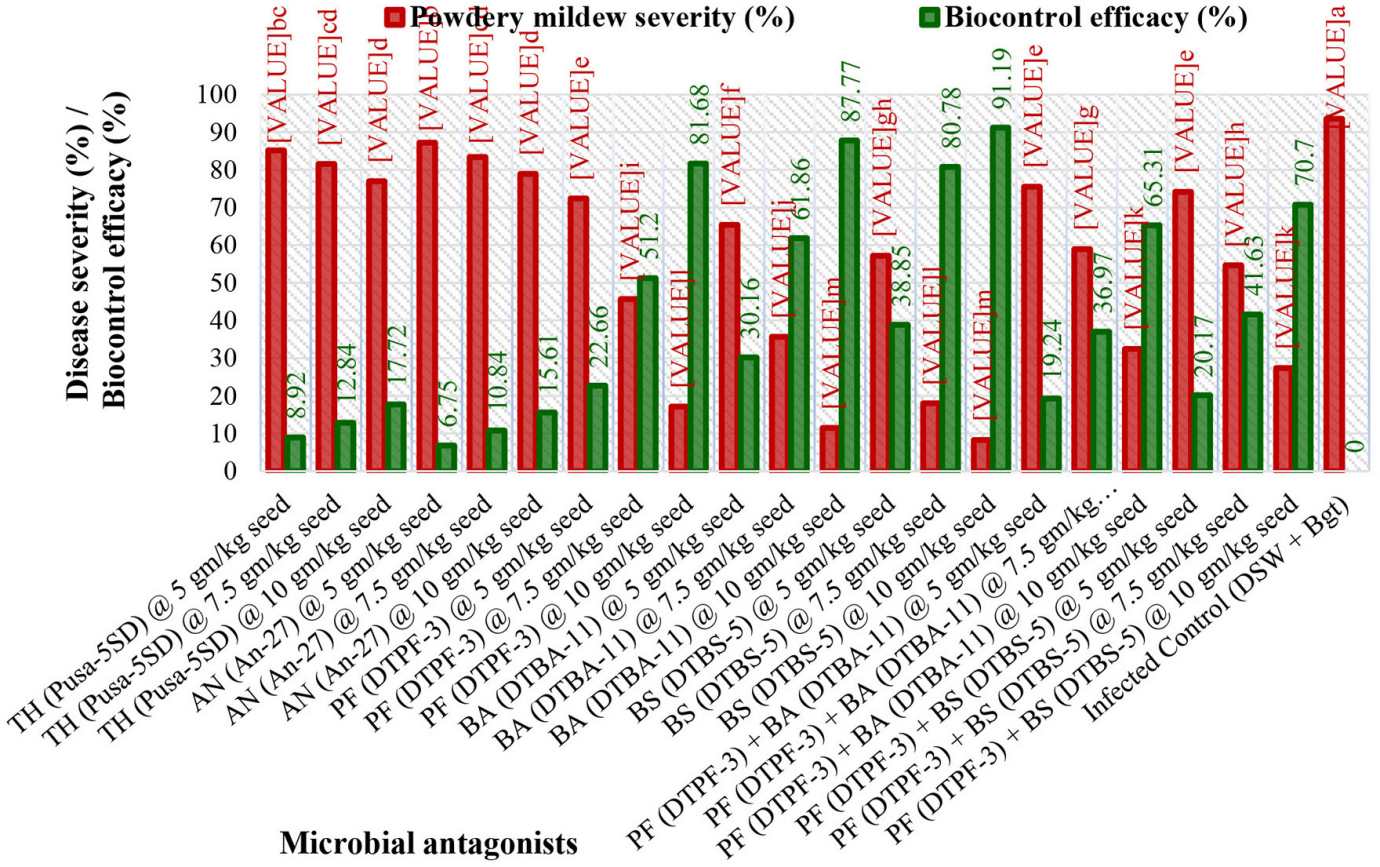
Effect of seed treatment with microbial antagonists and their combinations at different doses on disease severity and biological control efficacy in wheat against powdery mildew pathogens under greenhouse conditions. [TH (Pusa-5SD): *Trichoderma harzianum* strain Pusa-5SD; AN (An-27): *Aspergillus niger* strain An-27; PF (DTPF-3): *Pseudomonas fluorescens* strain DTPF-3; BA (DTBA-11): *Bacillus amyloliquefaciens* strain DTBA-11; and BS (DTBS-5): *Bacillus subtilis* strain DTBS-5]. DSW: sterile distilled water; *Bgt*: *Blumeria graminis* f. sp. *tritici*. The data are based on the mean of four replicates with 10 plants. The means followed by the same letters on the bar diagram are not significantly different, as determined by Duncan's Multiple Range Test at the 5% level.

**Figure 4:**
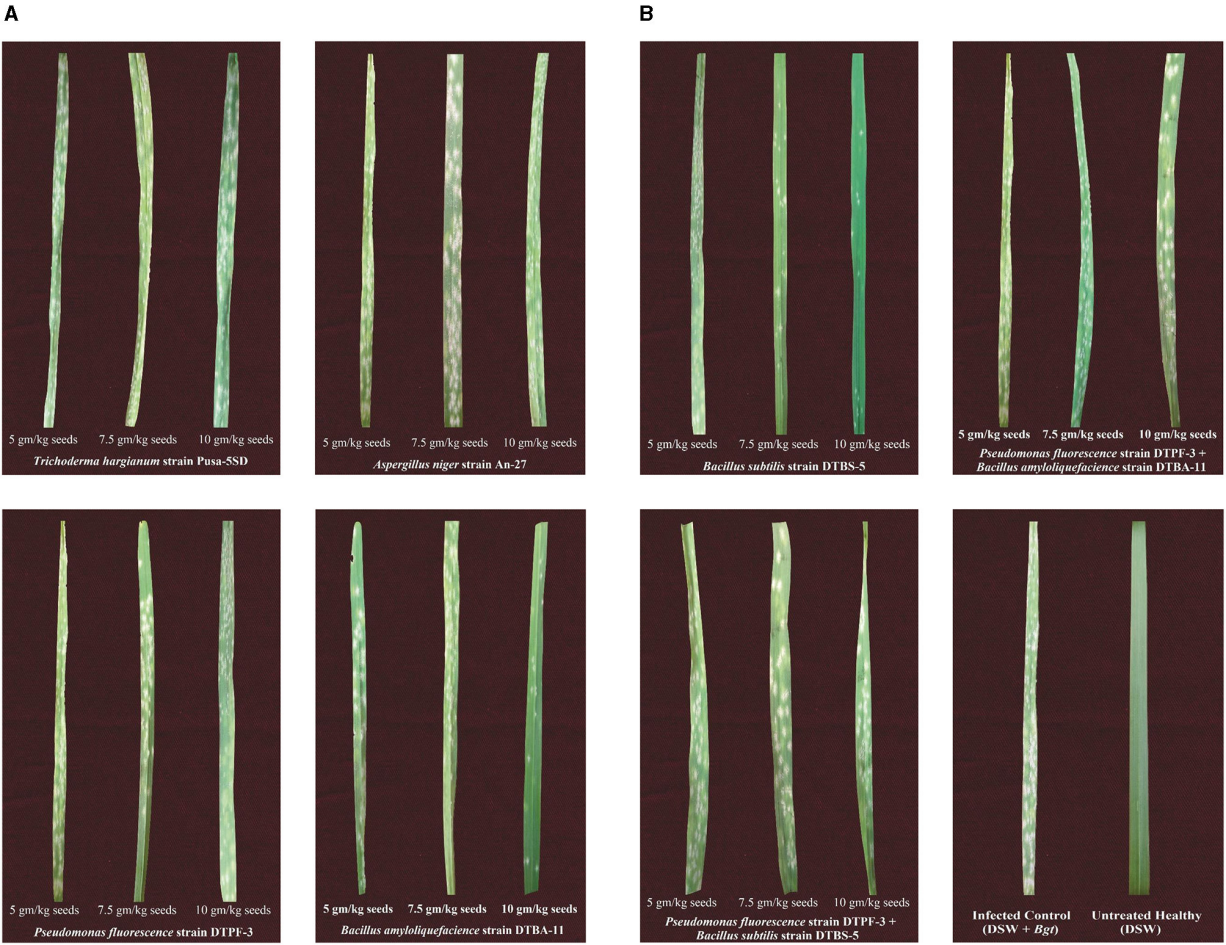
**(A)** Effect of seed treatment with different microbial antagonists and their combinations at different doses on disease severity in wheat against powdery mildew pathogens under greenhouse conditions. **(B)** Effect of seed treatment with different microbial antagonists and their combinations at different doses on disease severity in wheat against powdery mildew pathogens under greenhouse conditions.

The disease progress curves (DPCs) were determined by plotting mean disease severity against time (Figures 2–4). The DPCs obtained for the infected control followed a typical sigmoid curve. The seeds treated with the antagonist *BS*-DTBS-5 at 10 gm/kg showed slight disease progression, followed by BA-DTBA-11 and PF-DTPF-3 at the same dose, indicating higher effectiveness against the powdery mildew pathogen in wheat. In control pots, we observed a prolonged log phase (rise in disease severity) after 14 dpi. The seeds treated with consortia of *PF-*DTPF-3 + *BS-*DTBS-5 and *PF-DTPF-3* + *BA-*DTBA-11 showed a prolonged lag phase (low amount of disease) up to 21 dpi, which could be attributed to the moderate effectiveness of antagonists. The seeds treated with fungal antagonists, *T. harzianum* Pusa-5SD and *A. niger* An-27, exhibited continued log phase (disease progression) after 14–21 dpi, which indicated their ineffectiveness in controlling powdery mildew development on wheat.

#### Relative Area under the disease progress curve

**Figure 5:**
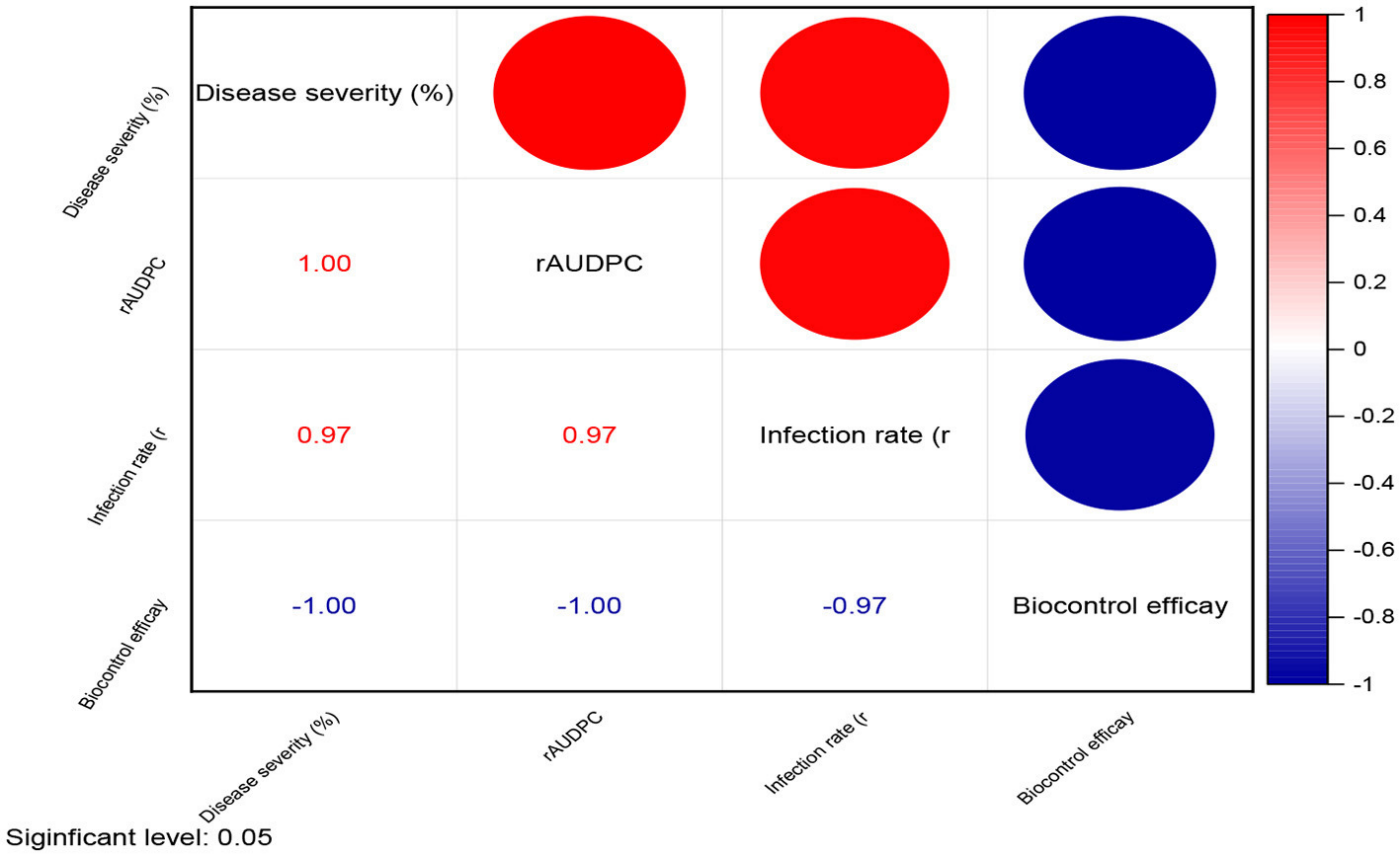
Correlation matrix of disease parameters and biocontrol efficacy in plant treatment studies [treatment dose of 10 g/kg seeds] against powdery mildew.

**Figure 6:**
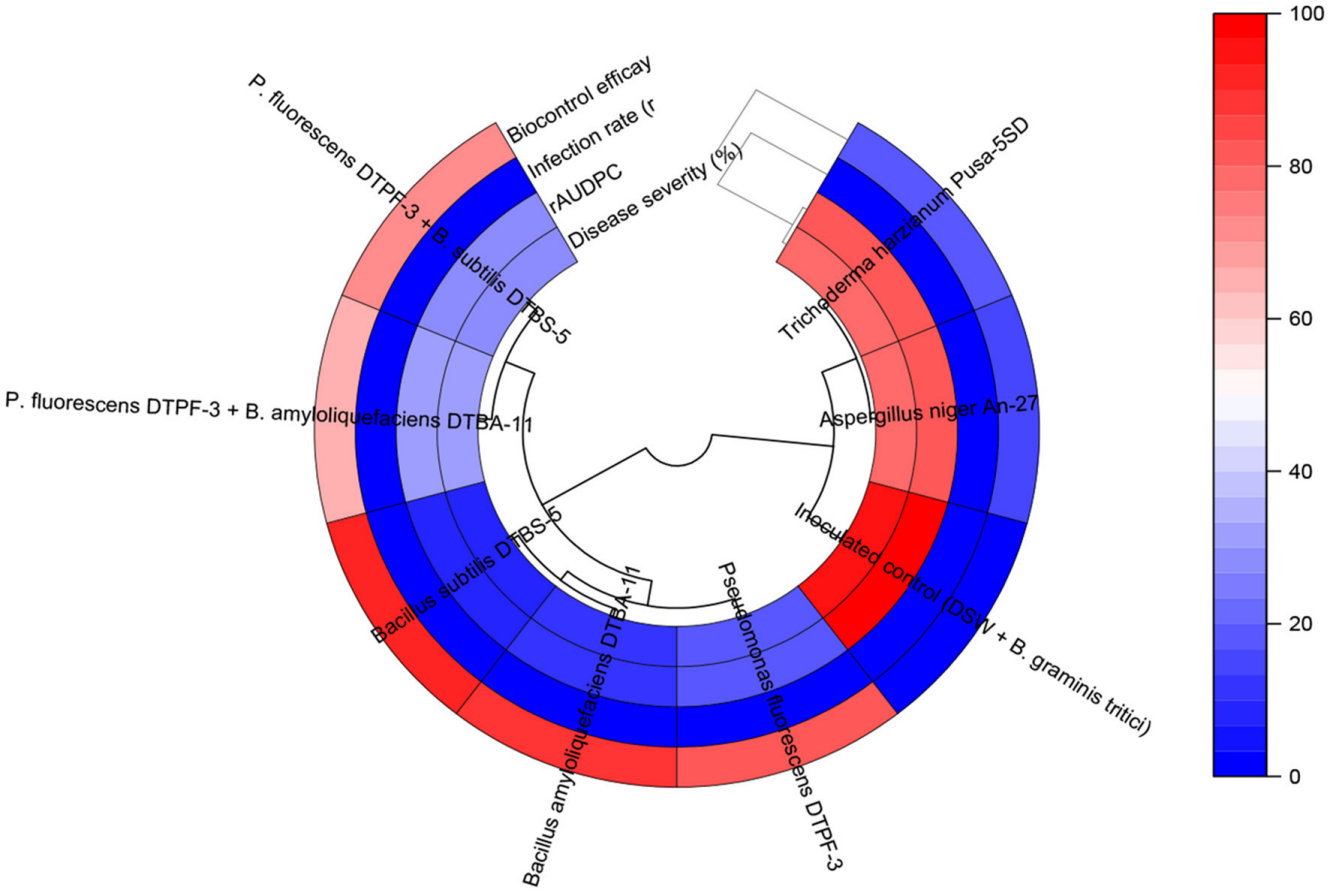
The circular heatmap of treatment effects on powdery mildew and biocontrol efficacy parameters.

The infected control exhibited the highest mean relative AUDPC (rAUDPC) value (100) compared to the treated groups (Supplementary Table 1). Among the treatments, the lowest mean rAUDPC value (6.88) was observed with the application of the antagonist *BS-*DTBS-5 at a rate of 10 g/kg seeds, followed by *BA-*DTBA-11 (10.15) and *PF-*DTPF-3 (16.82), indicating effective disease control by these antagonistic treatments. Additionally, mean rAUDPC values of 26.78 and 32.82 were recorded with the combinations of antagonists *PF-*DTPF-3 + *BS-*DTBS-5 and *PF-*DTPF-3 + *BA-*DTBA-11 at 10 g/kg seeds, respectively, showing moderate effectiveness against the disease (Figures 5, 6). The fungal antagonists, *viz., TH-*Pusa-5SD and *AN-*An-27, treated seeds at all three doses and recorded higher rAUDPC values (82.05–90.65) than other treatments, including the infected control. Due to the ineffectiveness of these fungal antagonistic treatments, the higher rAUDPC value showed higher disease severity, which is positively associated with the percent disease severity (Milus and Line, 1986).

#### Apparent infection rate (r)

**Figure 7:**
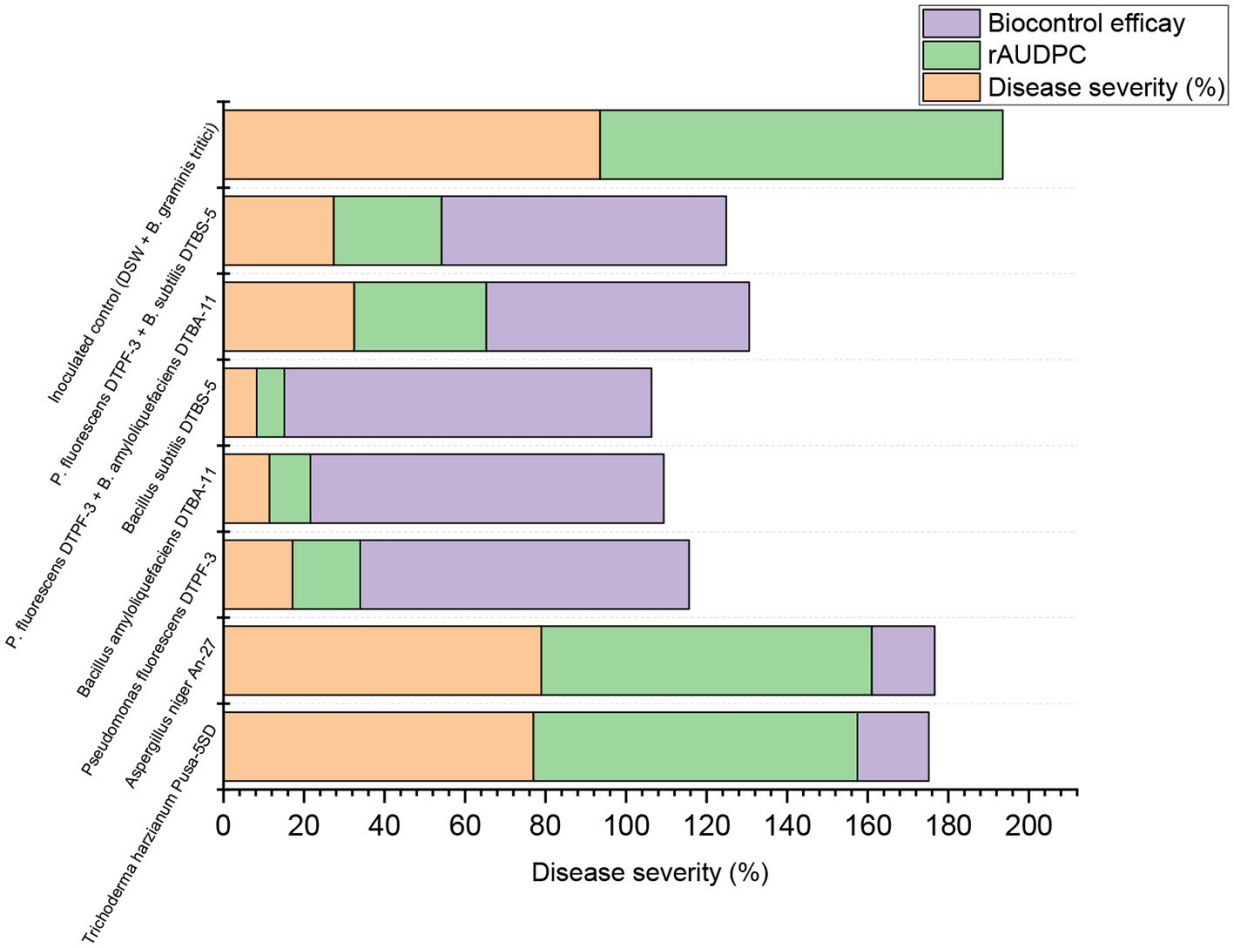
Stacked bar showing impact assessment of various treatments on plant disease severity and progression.

The apparent infection rate (r) of all tested microbial antagonists and their combinations was lower than that of the infected control, with a mean r-value of 0.39 (Supplementary Table 1). Specifically, *BS-*DTBS-5, followed by *BA-*DTBA-11 and *PF-*DTPF-3 at 10 g/kg seeds, consistently exhibited reduced disease severity, resulting in minimal disease progression over time, with mean r-values of 0.03, 0.06, and 0.07, respectively. Similarly, the combinations of *PF-*DTPF-3 + *BS-*DTBS-5 and *PF-DTPF-3* + *BA-*DTBA-11 at 10 g/kg seeds displayed moderately reduced disease severity, with mean r-values of 0.08 and 0.09, respectively. The fungal antagonists, such as *TH*-5SD and *AN-*An-27, treated at all three doses, exhibited the highest mean r-values (0.23–0.29), indicating ineffective control against the disease based on disease severity and rAUDPC values. In this study, treatments classified as highly effective biocontrol agents demonstrated infection rates of < 0.06, while those categorized as moderately effective displayed infection rates ranging from 0.07 to 0.09 concerning other disease parameters such as disease severity and rAUDPC (Figure 7).

### Expression of defense-related enzymes and biochemical activity changes

**Figure 8:**
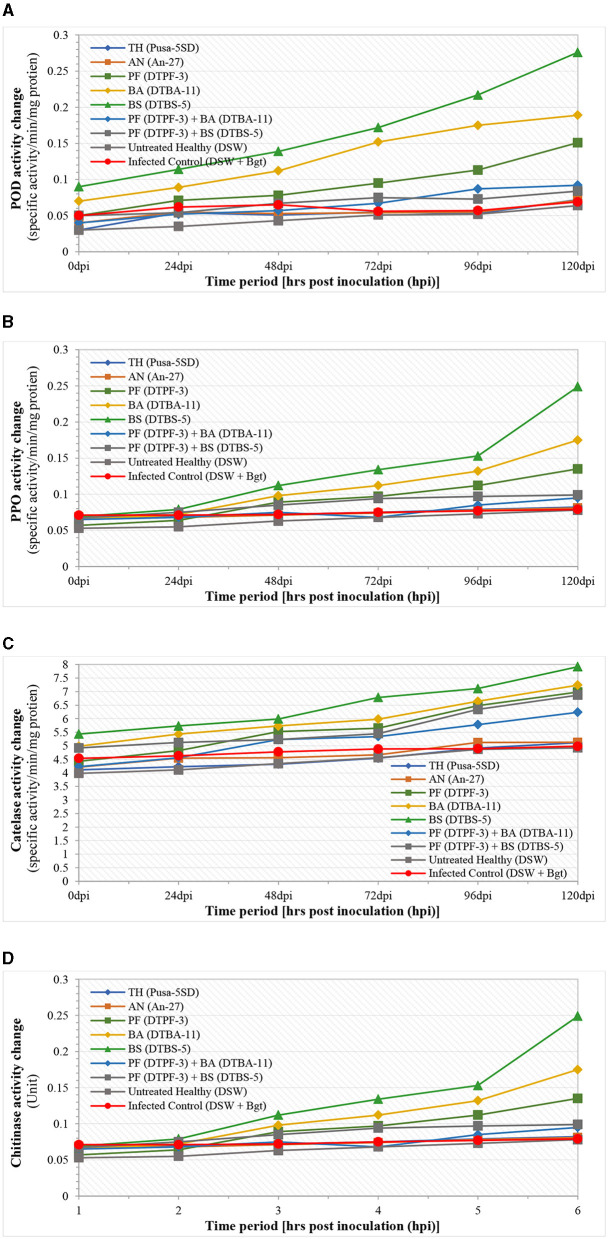
**(A)** Peroxidase (POD), **(B)** polyphenol oxidase (PPO), **(C)** catalase (CAT), and **(D)** chitinase activities in microbial antagonist-treated wheat tissues after infection with *B. graminis tritici* (*Bgt*). [TH (Pusa-5SD): *Trichoderma harzianum* strain Pusa-5SD; AN (An-27): *Aspergillus niger* strain An-27; PF (DTPF-3): *Pseudomonas fluorescens* strain DTPF-3; BA (DTBA-11): *BA-*DTBA-11; and BS (DTBS-5): *BS-*DTBS-5]. DSW: sterile distilled water.

**Figure 9:**
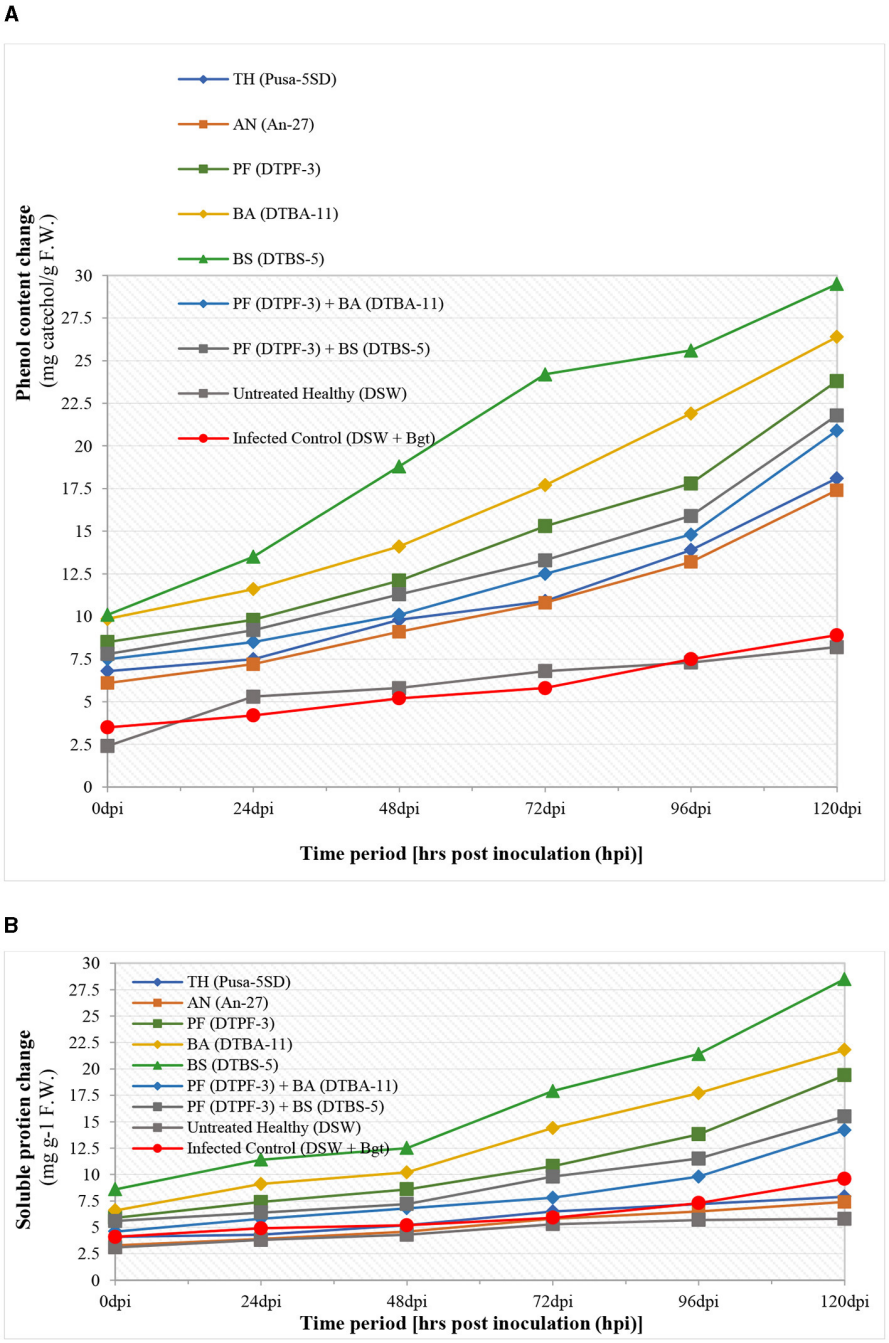
Biochemicals [total phenolic content **(A)** and soluble protein **(B)**] activity changes in microbial antagonist-treated wheat tissues after infection with *B. graminis tritici* (*Bgt*). [TH (Pusa-5SD): *Trichoderma harzianum* strain Pusa-5SD; AN (An-27): *Aspergillus niger* strain An-27; PF (DTPF-3): *Pseudomonas fluorescens* strain DTPF-3; BA (DTBA-11): *Bacillus amyloliquefaciens* strain DTBA-11; and BS (DTBS-5): *Bacillus subtilis* strain DTBS-5]. DSW: sterile distilled water.

The different defense-related enzymes and biochemical tests were assessed spectrophotometrically in the antagonist-treated wheat tissues after infection with *B. graminis tritici* (*Bgt*) (Figures 8, 9). The peroxidase enzyme activity was found to be highest in the wheat treated with the antagonists *BS-*DTBS-5 and *Bgt* after 120 h of infection (0.276 specific activity/min/mg protein), followed by *BA-*DTBA-11 (0.189 specific activity/min/mg protein) and *PF-*DTPF-3 (0.151 specific activity/min/mg protein). Comparatively, a moderate increase in POD activity was also noticed in the wheat treated with combinations of antagonists *PF-*DTPF-3 + *BS-*DTBS-5 (0.092 specific activity/min/mg protein) and *PF-*DTPF-3 + *BA-*DTBA-11 (0.084 specific activity/min/mg protein) and *Bgt* after 120 hpi. Apart from the untreated healthy and the infected control, no significant increase in POD activity was detected in the wheat treated with the fungal antagonists *TH-*Pusa-5SD (0.072 specific activity/min/mg protein) and *AN-*An-27 (0.071 specific activity/min/mg protein) and *Bgt* after 120 hpi. Polyphenol oxidase (PPO) activity in wheat was found to be comparatively higher (0.249 specific activity/min/mg protein) in the plants treated with bioagent *BS-*DTBS-5, followed by *BA-*DTBA-11 (0.175 specific activity/min/mg protein) and *PF-*DTPF-3 (0.135 specific activity/min/mg protein) and *Bgt* after 120 h of infection (Figure 8). A slight increase in PPO activity was also noted in the wheat treated with mixtures of *PF-*DTPF-3 + *BS*-DTBS-5 (0.082 specific activity/min/mg protein) and *PF*-DTPF-3 + *BA-*DTBA-11 (0.081 specific activity/min/mg protein) and *Bgt* after 120 hpi. Catalase (CAT) activity in the wheat treated with *BS-*DTBS-5 was the highest and remained at high levels (7.918 specific activity/min/mg protein) up to 120 h after *Bgt* infection, followed by *BA-*DTBA-11 (7.241 specific activity/min/mg protein) and *PF-*DTPF-3 (6.982 specific activity/min/mg protein). Chitinase activity in the wheat seeds treated with *BS-*DTBS-5 showed peak activity of chitinase (2.9 units) at 48 hpi and maintained high levels (6.9 units) up to 120 hpi with *Bgt*, followed by *BA-*DTBA-11 (5.7 units) and *PF-*DTPF-3 (5.2 units). A reasonable increase in chitinase activity was also observed in the wheat treated with combinations of antagonists *PF-*DTPF-3 + *BS-*DTBS-5 (4.8 units) and *PF-*DTPF-3 + *BA-*DTBA-11 (4.6 units) up to 120 hpi with *Bgt* pathogen. Total phenol content in the wheat treated with *BS-*DTBS-5 increased (13.5 mg catechol/g F.W.) just after 24 h of pathogen infection and maintained at high levels (29.5 mg catechol/g F.W.) up to 120 hpi with *Bgt*, followed by *BA-*DTBA-11 (26.4 mg catechol/g F.W.) and *PF-*DTPF-3 (23.8 mg catechol/g F.W.) (Figure 9). Phenol content was also found to be moderately increased in the wheat treated with combinations of antagonists *PF-*DTPF-3 + *BS-*DTBS-5 (21.8 mg catechol/g F.W.) and *PF*-DTPF-3 + *BA-*DTBA-11 (20.9 mg catechol/g F.W.) up to 120 hpi with *Bgt* pathogen.

Soluble protein content in wheat was also found to be comparatively higher (28.5 mg g^−1^ F.W.) in the plants treated with bioagent *BS-*DTBS-5, followed by *BA-*DTBA-11 (21.4 mg g^−1^ F.W.) and *PF-*DTPF-3 (19.4 mg g^−1^ F.W.) and *Bgt* after 120 h of infection. However, soluble protein content was also noted to increase moderately in the wheat treated with blends of *PF-*DTPF-3 + *BS-*DTBS-5 (15.5 mg g^−1^ F.W.) and *PF-*DTPF-3 + *BA-*DTBA-11 (14.2 mg g^−1^ F.W.) and *Bgt* after 120 hpi. In this study, significantly higher levels of defense-related enzymes (POD, PPO, catalase, and chitinase) and biochemicals (total phenolic content and soluble protein) activity changes were noticed in the wheat seeds treated with the antagonist *BS-*DTBS-5, followed by *BA-*DTBA-11 and *PF-*DTPF-3 at 10 g/kg seeds. Antagonistic combinations were also found to moderately induce resistance, while no significant results were noticed with the seed treatment with fungal antagonists after *Bgt* infection. The observed increase in defense enzyme activities such as POD, PPO, CAT, and chitinase, along with higher phenolic and soluble protein contents, suggests that microbial antagonists play a crucial role in priming the plant's innate immune response. Specifically, the 45% increase in POD activity and the 50% increase in CAT activity highlight the effectiveness of *Bacillus subtilis* strain DTBS-5 and *Bacillus amyloliquefaciens* strain DTBA-11, respectively, in managing the plant's oxidative stress. The 30% increase in phenolic content and the 25% increase in soluble protein content further support the role of these biocontrol agents in enhancing the biochemical defense of wheat plants against *B. graminis tritici*.

### Plant growth promotion attributes

**Figure 10:**
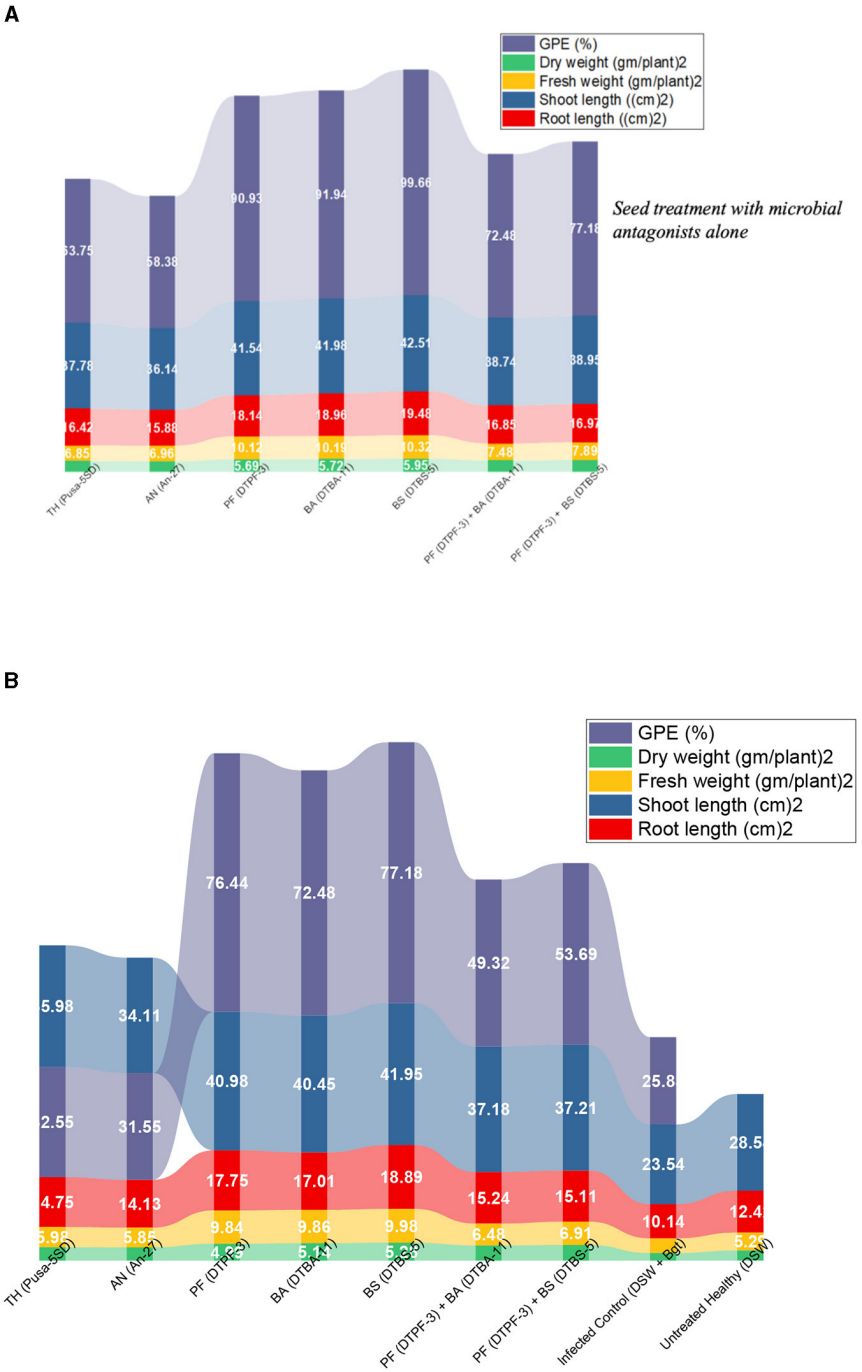
Quantitative variables (root length, shoot length, GPE, and plant weight) grouped by different treatments of microbial antagonists without **(A)** pathogen *Bgt* challenged and with **(B)** pathogen *Bgt* challenged plants and their growth parameters and germination efficacy.

The plant growth promotion abilities were evaluated in the wheat seeds bioprimed with microbial antagonists, both alone and co-infected with *Bgt*, under greenhouse conditions. The results revealed significant variations in the wheat seeds treated with the tested microbial antagonists alone and in conjunction with *Bgt* for various vegetative traits, including plant length, fresh weight, dry weight, and growth promotion efficacy (Figures 10A, B, Supplementary Table 2). In the seeds treated solely with microbial antagonists, the highest mean root length of 19.48 cm and shoot length of 42.51 cm were observed with *BS-*DTBS-5, followed by *BA*-DTBA-11 (18.98 cm and 41.98 cm) and *PF-*DTPF-3 (18.14 cm and 41.54 cm). In the seeds treated with combinations of antagonists *PF-*DTPF-3 + *BS-*DTBS-5 and *PF-*DTPF-3 + *BA-*DTBA-11, themean root lengths were 16.97 cm and 16.85 cm and mean shoot lengths were 38.95 cm and 38.74 cm, respectively. The maximum mean plant dry weight (5.95 gm) was recorded with *BS-*DTBS-5, followed by *BA-*DTBA-11 (5.72 gm) and *PF-*DTPF-3 (5.69 gm). The antagonistic combinations of *PF-*DTPF-3 + *BS-*DTBS-5 and *PF-*DTPF-3 + *BA-*DTBA-11 also exhibited mean plant dry weights of 5.28 g and 5.14 g, respectively. The growth promotion efficacy (GPE) was significantly higher (99.66%) with *BS-*DTBS-5, followed by *BA-*DTBA-11 (91.94%) and *PF-*DTPF-3 (90.93%). The antagonistic combinations of *PF-*DTPF-3 + *BS-*DTBS-5 and *PF-*DTPF-3 + *BA-*DTBA-11 also exhibited GPEs of 77.18% and 72.48%, respectively. In the seed treatment with microbial antagonists and infected with *B. graminis tritici*, the maximum mean root length of 18.89 cm and shoot length of 41.95 cm were noticed in *BS-*DTBS-5, trailed by *BA-*DTBA-11 (17.01 cm and 40.45 cm) and *PF-*DTPF-3 (17.75 cm and 40.98 cm). The combinations of *PF-*DTPF-3 + *BS-*DTBS-5 and *PF-*DTPF-3 + *BA-*DTBA-11 gave mean root lengths of 15.11 cm and 15.24 cm, and mean shoot lengths of 37.21 cm and 37.18 cm, respectively. The maximum mean plant dry weight (5.28 gm) was recorded in *BS-*DTBS-5, followed by *BA-*DTBA-11 (5.14 gm) and *PF-*DTPF-3 (4.99 gm). The antagonistic mixtures of *PF-*DTPF-3 + *BS-*DTBS-5 and *PF-*DTPF-3 + *BA-*DTBA-11 were yielded mean plant dry weights of 4.58 g and 4.45 g, respectively. The GPE was higher (77.18%) in *BS-*DTBS-5, followed by *BA-*DTBA-11 (72.48%) and *PF-*DTPF-3 (76.44%). The antagonistic combinations of *PF-*DTPF-3 + *BS-*DTBS-5 and *PF-*DTPF-3 + *BA-*DTBA-11 also recorded GPEs of 53.69% and 49.32%, respectively.

In this research, the wheat treated with microbial antagonists alone showed no discernible variation in plant growth promotion activity. On the other hand, the wheat treated with microbial antagonists and infected with the *Bgt* pathogen showed notable differences in PGP characteristics.

## Discussion

The microbial antagonists, including *PF-*DTPF-3, *BA-*DTBA-11, *BS-*DTBS-5, *TH-*Pusa-5SD, and *AN-*An-27, had not been previously evaluated against the wheat powdery mildew pathogen. However, they were chosen for our investigation based on the biocontrol potential that different researchers have documented in other pathosystems (Mondal et al., 2000; Sheroze et al., 2003; Dubey et al., 2007; Anand and Reddy, 2009; Liu et al., 2009; Singh et al., 2010; Hui et al., 2013; Tan et al., 2013; Meena et al., 2017; Sánchez-Montesinos et al., 2021; Manzar et al., 2022; Kashyap and Manzar, 2023). In this study, *BS-*DTBS-5 emerged as the most effective biocontrol agent, which aligns with the reports by Singh et al. (2010). The synergistic effects of *B. subtilis* and *B. amyloliquefaciens* have been observed to enhance bioefficacy and decrease the incidence of black rot caused by *Xanthomonas campestris* pv. *campestris*. The biocontrol efficacy and plant growth promotion abilities of bacterial antagonists, specifically *BA-*DSBA-11 and DSBA-12, were assessed against the bacterial wilt of tomato cv. Pusa Ruby under greenhouse conditions. It was observed that plants treated with *BA-*DSBA-12 exhibited superior growth-promoting efficacy compared to those treated with *B. pumilus* MTCC-7092 (Singh et al., 2016). Multiple microbial antagonists, such as *Pseudomonas fluorescens* PDS1, *Bacillus subtilis* BDS1, *Bacillus cereus* UK4, *Bacillus amyloliquefaciens* UK2, and *Bacillus subtilis* KA9, have been reported for their antagonistic and growth promotion activity against Chilli Wilt disease caused by *Ralstonia solanacearum* (Kashyap et al., 2021, 2022; Kashyap and Manzar, 2023). Our studies showed that *BA-*DTBA-11 and *PF-*DTPF-3 at 10 g/kg seeds significantly reduced PM severity by 11.44% and 17.14%, respectively, with mean biocontrol efficacies of 87.77% and 81.68%. A similar finding was recorded in a case study of rust, where biocontrol agents *P. fluorescens* strain PF-1 (5 gm/L water) and *B. subtilis* strain BS-1 (5 gm/L water) were found to be moderately effective in controlling wheat stripe rust by 41.83% and 39.92%, respectively (Singh et al., 2016). Our findings align with those of Gao et al. (2015), who demonstrated the biocontrol potential of four strains of endophytic bacteria (E1R-j, E1R-h, ECL5, and Em7) against wheat powdery mildew, *Bgt*. Additionally, they reported that spraying E1R-j 24 h before *Bgt* inoculation inhibited conidial germination and appressorial development, with a 42.7% reduction in appressorial development and a 43.3% reduction in conidial germination compared to the water control. *Bacillus* organisms, being gram-positive bacteria, have many advantages that make them a potential candidate for the biological control of plant diseases. *Bacillus* can produce spores that resist UV light and adverse conditions, qualifying them for effective commercial bioformulation (Raaijmakers et al., 2002). Gram-negative bacteria from the genera *Pseudomonas* and *Erwinia* have also gained significant recognition as biological control agents (Cartwright et al., 1995; Braun-Kiewnick et al., 2000; Shoda, 2000; Slininger et al., 2000; Costa et al., 2001). *B. amyloliquefaciens* strain YN201732, a beneficial endophyte isolated from tobacco, was found effective in controlling tobacco powdery mildew and inducing systemic resistance. It was also found to be effective in inducing polyphenol oxidase and chitinase activity changes in tobacco plants infected with powdery mildew (Santoyo et al., 2012; Kannojia et al., 2019). Single nucleotide polymorphisms (SNPs) obtained from *Pseudomonas putida* (PpFT1) and *B. subtilis* (BsBN3) at 50, 100, and 150 ppm were found to be most effective in reducing powdery mildew severity by 83.3, 89.7, 84.6, and 91.0%, respectively. SNPs obtained from *T. harzianum* at 150 ppm have been reported to reduce PM severity by 82.0% (Farhat et al., 2018). The wheat crop treated with microbial antagonists *B. subtilis* strain DTBS-5 and pathogen Bgt showed greater elicitation of defense-related enzymes (POD, PPO, CAT, and chitinase) and biochemicals (total phenolic and soluble protein content) at 120 hpi, followed by another antagonist *B. amyloliquefaciens* strain DTBA-11 and *P. fluorescens* strain DTPF-3. Similar elicitations of defense-related enzymes and biochemical activities were observed in wheat seed biopriming with a mixture of antagonists, *P. fluorescens* strain DTPF-3 + *B. subtilis* strain DTBS-5 and *P. fluorescens* strain DTPF-3 + *B. amyloliquefaciens* strain DTBA-11 at 120 hpi. The strain DTBS-5 likely produces specific elicitors or signaling molecules that are recognized by the wheat plant's innate immune system. Upon recognition, these signals trigger a cascade of defense responses, including the activation of enzymes such as POD, PPO, CAT, and chitinase, which play crucial roles in strengthening the plant's defense barriers against pathogens. The conclusions drawn from our study align with the research conducted earlier (Manzar et al., 2021; Kashyap et al., 2022), indicating that seed biopriming utilizing *Bacillus* sp. or biocontrol agents leads to the elicitation of defense-related enzymes such as PAL, peroxidase, and polyphenol oxidase within 48 h, thereby priming resistance against *Bgt*. Specifically, our findings are consistent with the activities of polyphenol oxidase and peroxidase in the tomato plants treated with *B. amyloliquefaciens* SQRT3 strain and *R. solanacearum* compared to other treatments, which is consistent with previous reports (Shishkoff and McGrath, 2002; Punja and Utkhede, 2003). Apart from the untreated healthy and the infected control, no significant increase in defense-related enzymes or biochemical activity changes were noticed in the wheat seeds treated with the fungal antagonists *T. harzianum* strain Pusa-5SD and *A. niger* strain An-27. This variation in efficacy across microbial antagonists emphasizes the complexities of plant-microbe interactions and the need to select appropriate biocontrol agents based on their mode of action and compatibility with the host plant's defensive system. The results highlight the potential of using microbial antagonists to enhance crop resilience through induced resistance, offering a sustainable alternative to chemical pesticides (Kashyap et al., 2022). This study observed significantly higher levels of defense-related enzymes and biochemical activity changes in *B. subtilis* strain DTBS-5-treated plants, followed by *B. amyloliquefaciens* strain DTBA-11 and *P. fluorescens* strain DTPF-3. Comparatively, antagonistic mixtures were also found to be moderate in terms of induced resistance and biochemical changes, whereas fungal antagonists showed non-significant results (Singh Vaibhav, 2017; Farhat et al., 2018; Manzar et al., 2021, 2022; Jatoth et al., 2024).

The observed increase in defense enzyme activities such as peroxidase (POD), polyphenol oxidase (PPO), catalase (CAT), and chitinase, along with higher phenolic and soluble protein contents, suggests that microbial antagonists play a crucial role in priming the plant's innate immune response. This priming effect is consistent with the results of previous studies, which have demonstrated that microbial antagonists can induce systemic resistance in plants by activating various defense mechanisms (Van Loon et al., 1998; Pieterse et al., 2014). The 45% increase in POD activity and the 50% increase in CAT activity highlight the effectiveness of *Bacillus subtilis* strain DTBS-5 and *Bacillus amyloliquefaciens* strain DTBA-11, respectively, in bolstering the plant's oxidative stress. Peroxidase and catalase are key enzymes in the plant's antioxidant defense system, which helps mitigate the damage caused by reactive oxygen species (ROS) generated during pathogen attacks. The enhancement of these enzyme activities by biocontrol agents has been well documented. For instance, Zhang et al. (2007) reported that *Bacillus subtilis* increased POD and CAT activities in cucumbers, leading to improved resistance against Fusarium wilt. The 30% rise in phenolic content and the 25% increase in soluble protein content further support the role of these biocontrol agents in enhancing the biochemical defense arsenal of wheat plants against *Blumeria graminis* f. sp. *tritici* (Bgt). Phenolic compounds are known to contribute to plant defense by strengthening cell walls and acting as antimicrobial agents. Increased phenolic content following treatment with microbial antagonists has been reported in various crops. Aziz et al. (2003) observed elevated phenolic levels in grapevine treated with *T. harzianum*, which correlated with enhanced resistance to *Botrytis cinerea*.

Similarly, soluble proteins, including pathogenesis-related (PR) proteins, play a critical role in plant defense mechanisms. PR proteins, such as chitinases, degrade the cell walls of fungal pathogens, thereby inhibiting their growth and proliferation. The increase in soluble protein content observed in this study is in line with previous findings from the study by Benhamou and Brodeur (1998), who demonstrated that *T. harzianum* induced the accumulation of PR proteins in cucumber, enhancing its resistance to *Pythium ultimum* (Benhamou and Brodeur, 1998; Van Loon et al., 1998; Aziz et al., 2003; Zhang et al., 2007; Pieterse et al., 2014; Singh Vaibhav, 2017).

## Conclusion

The study concludes that treating wheat seeds with microbial antagonists at 10 g/kg significantly mitigates the severity of powdery mildew (PM), with *B. subtilis* strain DTBS-5 emerging as the most effective. This strain not only reduced PM severity to the lowest observed level of 8.24% but also achieved the highest biological control efficacy (BCE) at 91.19%. Close behind in effectiveness were *B. amyloliquefaciens* (DTBA-11) and *P. fluorescens* (DTPF-3), demonstrating considerable disease control capabilities. Despite their potential, fungal antagonists such as *T. harzianum* (Pusa-5SD) and *A. niger* (An-27) were ineffective against PM. The study also highlighted the ability of microbial antagonists to induce defense mechanisms in wheat, with notable increases in defense-related enzymes and biochemicals following treatment with the most effective strains. Microbial antagonists promoted plant growth, with *B. subtilis* strain DTBS-5 enhancing root and shoot length and plant dry weight. This research highlights the potential of microbial antagonists as a dual-function tool for controlling wheat PM and promoting plant growth, presenting a viable, eco-friendly alternative to chemical fungicides and bacterial antagonists that outperform fungal antagonists for foliar disease management.

## Data availability statement

The original contributions presented in the study are included in the article/Supplementary material, further inquiries can be directed to the corresponding authors.

## Author contributions

KC: Writing – original draft, Methodology, Investigation, Conceptualization. VS: Writing – original draft, Supervision, Project administration, Methodology, Investigation, Funding acquisition, Data curation, Conceptualization. AK: Writing – review & editing, Visualization, Software, Formal analysis, Data curation. AP: Validation, Writing – review & editing, Methodology. KS: Formal analysis, Writing – review & editing, Methodology. DY: Validation, Methodology, Writing – review & editing, Data curation. NM: Software, Formal analysis, Data curation, Writing – review & editing. DK: Methodology, Investigation, Funding acquisition, Writing – review & editing. LP: Project administration, Data curation, Writing – review & editing. MS: Writing – review & editing, Supervision, Resources, Funding acquisition.
