# Supplementary material for: Evaluating the efficacy of microbial antagonists in inducing resistance, promoting growth, and providing biological control against powdery mildew in wheat
Source: Front Microbiol. 2024 Jul 23;15:1419547. doi: 10.3389/fmicb.2024.1419547 (PMC11304452; doi:10.3389/fmicb.2024.1419547)
Supplement: Supplementary file 1 [file Table_1.docx]

| **Treatment category** | **Dose**  **(gm/kg seeds)** | **Disease parameters^*^** | | | **Biocontrol efficay**  **(%)** |
| --- | --- | --- | --- | --- | --- |
|  |  | **Disease severity (%)** | **rAUDPC** | **Infection rate (*r*)** |  |
| *Trichoderma harzianum* Pusa-5SD | 5 | 85.21^bc^ | 88.60^b^ | 0.27^bc^ | 8.92 |
|  | 7.5 | 81.54^cd^ | 84.77^c^ | 0.25^bc^ | 12.84 |
|  | 10 | 76.98^d^ | 80.46^de^ | 0.23^bc^ | 17.72 |
| *Aspergillus niger* An-27 | 5 | 87.24^b^ | 90.65^b^ | 0.29^b^ | 6.75 |
|  | 7.5 | 83.41^cd^ | 86.83^bc^ | 0.26^bc^ | 10.84 |
|  | 10 | 78.95^d^ | 82.05^ef^ | 0.24^bc^ | 15.61 |
| *Pseudomonas fluorescens* DTPF-3 | 5 | 72.35^e^ | 75.63^f^ | 0.21^c^ | 22.66 |
|  | 7.5 | 45.65^i^ | 45.80^j^ | 0.09^defg^ | 51.20 |
|  | 10 | 17.14^l^ | 16.82^m^ | 0.06^efg^ | 81.68 |
| *Bacillus amyloliquefaciens* DTBA-11 | 5 | 65.34^f^ | 68.54^g^ | 0.14^d^ | 30.16 |
|  | 7.5 | 35.68^j^ | 35.93^k^ | 0.09^defg^ | 61.86 |
|  | 10 | 11.44^m^ | 10.15^n^ | 0.05f^g^ | 87.77 |
| *Bacillus subtilis* DTBS-5 | 5 | 57.21^gh^ | 59.86^hi^ | 0.12^def^ | 38.85 |
|  | 7.5 | 17.98^l^ | 18.36^m^ | 0.07^defg^ | 80.78 |
|  | 10 | 8.24^m^ | 6.88^n^ | 0.03^g^ | 91.19 |
| *P. fluorescens* DTPF-3 + *B. amyloliquefaciens* DTBA-11 | 5 | 75.55^e^ | 78.67^ef^ | 0.22^bc^ | 19.24 |
|  | 7.5 | 58.97^g^ | 62.63^h^ | 0.13^de^ | 36.97 |
|  | 10 | 32.45^k^ | 32.82^k^ | 0.09^defg^ | 65.31 |
| *P. fluorescens* DTPF-3 + *B. subtilis* DTBS-5 | 5 | 74.18^e^ | 77.55^ef^ | 0.22^bc^ | 20.17 |
|  | 7.5 | 54.61^h^ | 57.21^i^ | 0.11^def^ | 41.63 |
|  | 10 | 27.41^k^ | 26.78^l^ | 0.07^defg^ | 70.70 |
| Fungicide check (+ *B. graminis tritici*) |  |  |  |  |  |
| Inoculated control (DSW + *B. graminis tritici*) | - | 93.56^a^ | 100^a^ | 0.39^a^ | 0 |
| Untreated healthy (DSW) | - | - | - | - | - |

**Supplementary Table 1 Effect of microbial treatments on disease parameters and** **biocontrol efficacy in *B. graminis tritici* inoculated wheat cv. PBW 343 in the greenhouse.**

^*^The data are based on mean of four replicates with ten plants. Means followed by the same letters in each column is not significantly different as determined by Duncan’s Multiple Range Test at 5% level.

**Supplementary Table 2. Growth promotion effect (GPE) of seed treated microbial antagonists alone and in infected with powdery mildew pathogen, *B. graminis tritici* (*Bgt*) on biomass of wheat plant grown under greenhouse conditions.**

| **Treatment**^1^ | **Plant length (cm)**^2^ | | **Fresh weight (gm/plant)**^2^ | **Dry weight (gm/plant)**^2^ | **GPE (%)** |
| --- | --- | --- | --- | --- | --- |
|  | **Root** | **Shoot** |  |  |  |
| *Seed treatment with microbial antagonists alone* | | | | | |
| TH (Pusa-5SD) | 16.42^abcd^ | 37.78^bcde^ | 6.85^bcd^ | 4.88^abc^ | 63.75 |
| AN (An-27) | 15.88^abcd^ | 36.14^de^ | 6.96^bcd^ | 4.72^abc^ | 58.38 |
| PF (DTPF-3) | 18.14^abc^ | 41.54^a^ | 10.12^a^ | 5.69^ab^ | 90.93 |
| BA (DTBA-11) | 18.96^ab^ | 41.98^a^ | 10.19^a^ | 5.72^a^ | 91.94 |
| BS (DTBS-5) | 19.48^a^ | 42.51^a^ | 10.32^a^ | 5.95^a^ | 99.66 |
| PF (DTPF-3) +  BA (DTBA-11) | 16.85^abcd^ | 38.74^abcd^ | 7.48^bc^ | 5.14^abc^ | 72.48 |
| PF (DTPF-3) +  BS (DTBS-5) | 16.97^abcd^ | 38.95^abcd^ | 7.89^b^ | 5.28^abc^ | 77.18 |
| *Seed treatment with microbial antagonists + Bgt infection* | | | | | |
| TH (Pusa-5SD) | 14.75^cde^ | 35.98^de^ | 5.98^cde^ | 3.95^bcd^ | 32.55 |
| AN (An-27) | 14.13^de^ | 34.11^e^ | 5.85^cde^ | 3.91^cd^ | 31.55 |
| PF (DTPF-3) | 17.75^abcd^ | 40.98^ab^ | 9.84^a^ | 4.99^abc^ | 76.44 |
| BA (DTBA-11) | 17.01^abcd^ | 40.45^ab^ | 9.86^a^ | 5.14^abc^ | 72.48 |
| BS (DTBS-5) | 18.89^ab^ | 41.95^a^ | 9.98^a^ | 5.28^abc^ | 77.18 |
| PF (DTPF-3) +  BA (DTBA-11) | 15.24^bcde^ | 37.18^cde^ | 6.48^bcd^ | 4.45^abcd^ | 49.32 |
| PF (DTPF-3) +  BS (DTBS-5) | 15.11^cde^ | 37.21^cde^ | 6.91^bcd^ | 4.58^abcd^ | 53.69 |
| Infected Control  (DSW + *Bgt*) | 10.14^f^ | 23.54^g^ | 4.35^e^ | 2.21^e^ | 25.83 |
| Untreated Healthy (DSW) | 12.42^ef^ | 28.54^f^ | 5.29^de^ | 2.98^de^ | - |

^1^TH (Pusa-5SD): *Trichoderma harzianum* strain Pusa-5SD; AN (An-27): *Aspergillus niger* strain An-27; PF (DTPF-3): *Pseudomonas fluorescens* strain DTPF-3; BA (DTBA-11): *Bacillus amyloliquefaciens* strain DTBA-11 and BS (DTBS-5): *Bacillus subtilis* strain DTBS-5; DSW: Distilled Sterile Water; *Bgt*: *B. graminis* f. sp. *tritici*. ^2^The data are based on mean of four replicates with ten plants. Means followed by the same letters in each column is not significantly different as determined by Duncan’s Multiple Range Test at 5% level.
