# Supplementary material for: Evaluating the efficacy of microbial antagonists in inducing resistance, promoting growth, and providing biological control against powdery mildew in wheat
Source: Front Microbiol. 2024 Jul 23;15:1419547. doi: 10.3389/fmicb.2024.1419547 (PMC11304452; doi:10.3389/fmicb.2024.1419547)
Supplement: Supplementary file 3 [file Image_2.pdf]

Showing comparative efficacy of biological control agents (Treatments Dose @7.5 gm/kg seeds) against powdery mildew disease parameters *viz.* Disease severity (%), rAUDPC, Infection rate (r). The data are based on four times weekly disease observations and mean of four replicates with ten plants

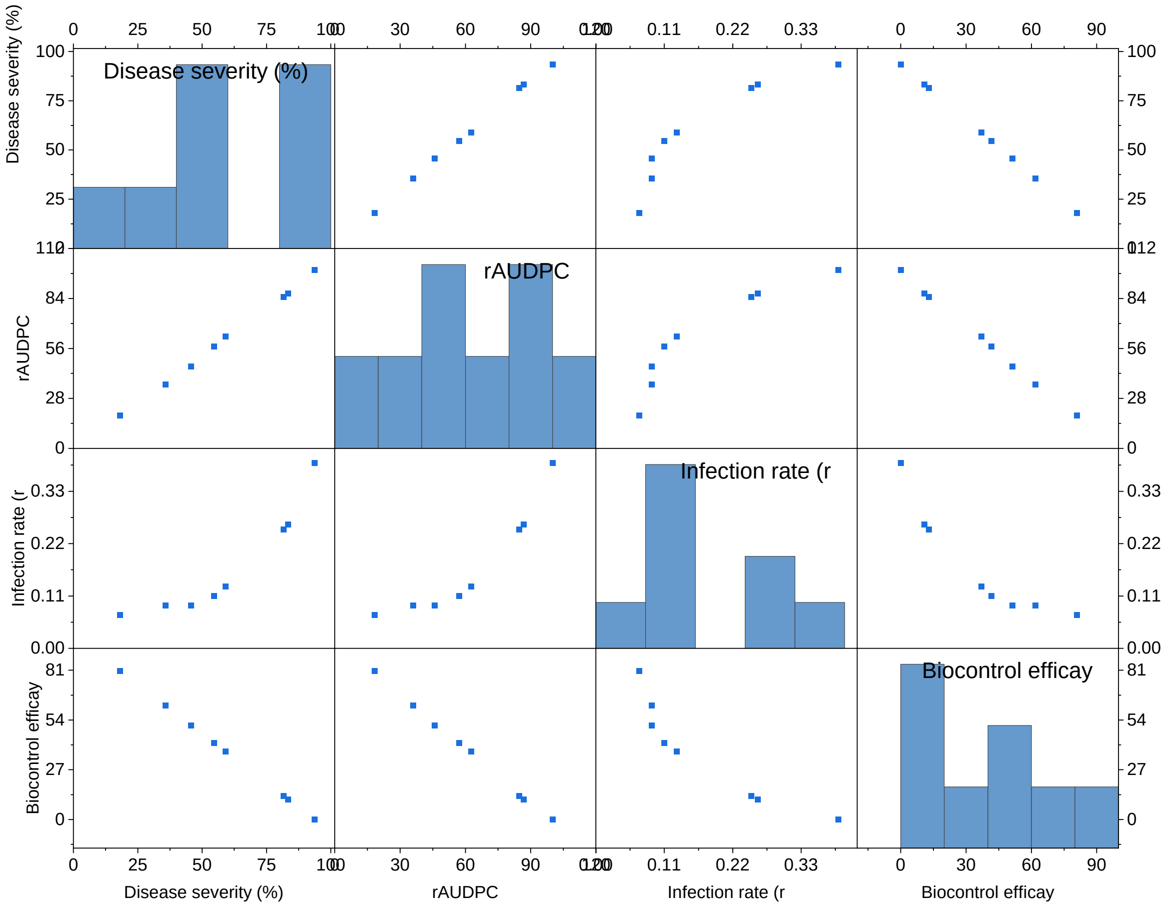

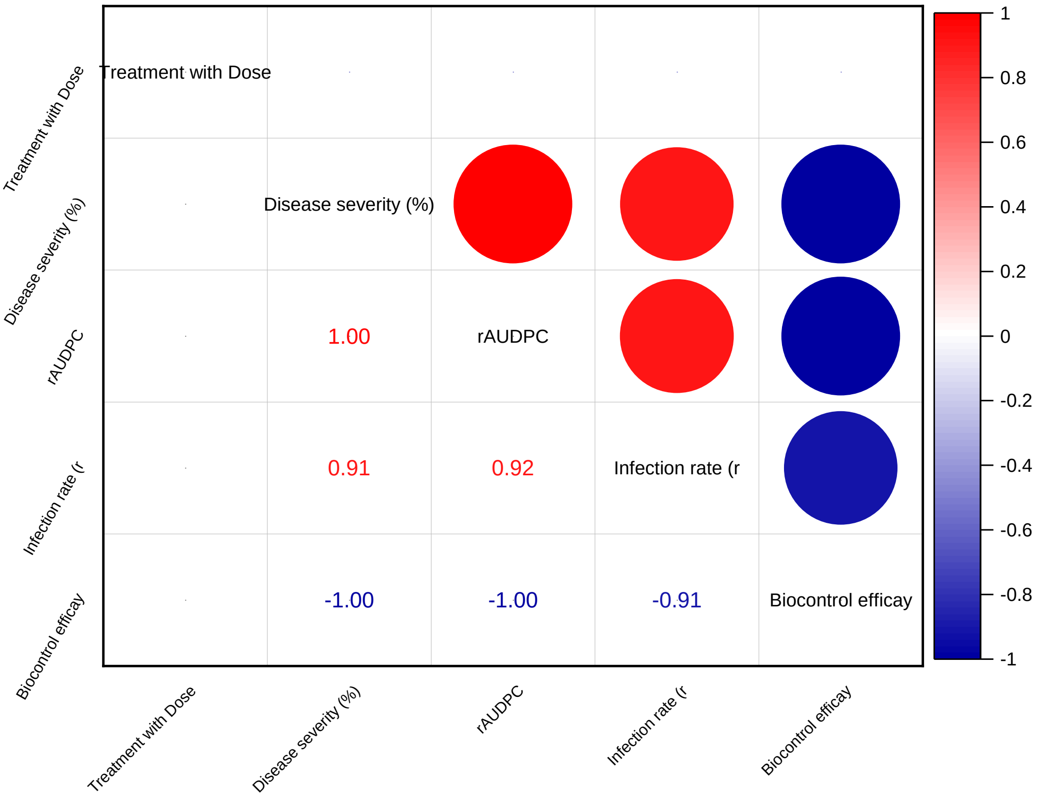

Siginficant level: 0.05

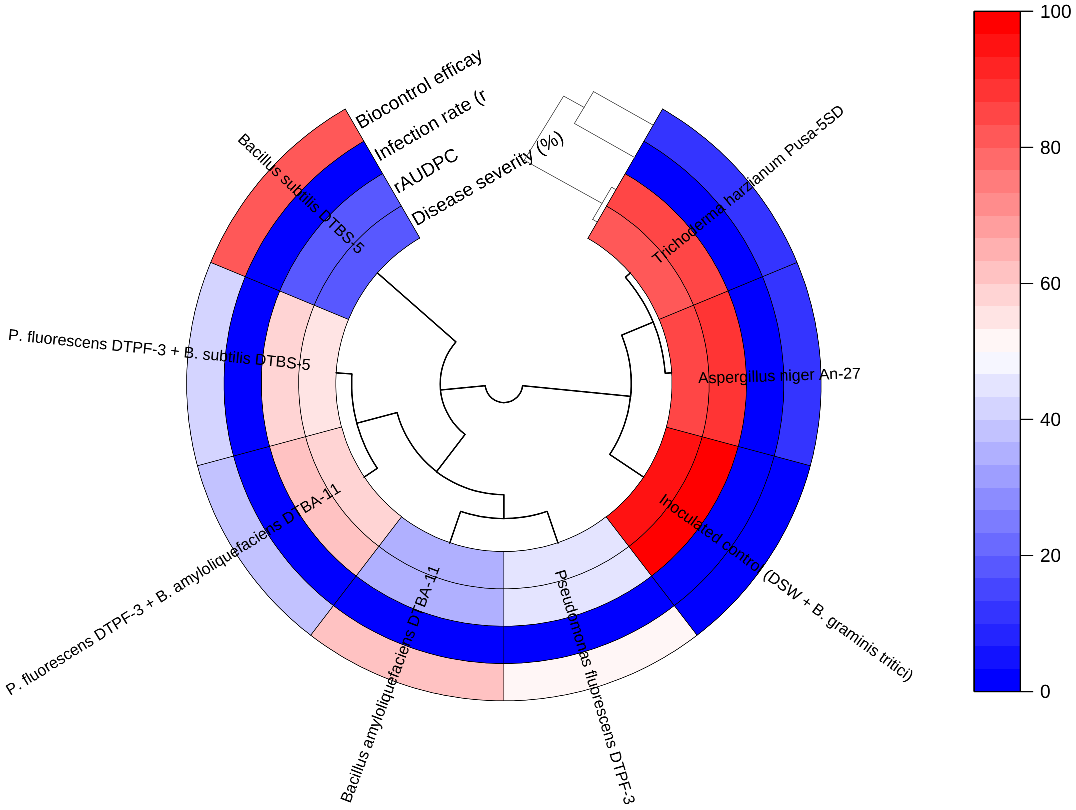

Biocontrol efficay
Infection rate (r
rAUDPC
Disease severity (%)
Trichoderma harzianum Pusa-5SD
Aspergillus niger An-27
Inoculated control (DSW + B. graminis tritici)
Pseudomonas fluorescens DTPF-3
Bacillus amyloliquefaciens DTBA-11
P. fluorescens DTPF-3 + B. amyloliquefaciens DTBA-11
P. fluorescens DTPF-3 + B. subtilis DTBS-5
Bacillus subtilis DTBS-5
100
80
60
40
20
0

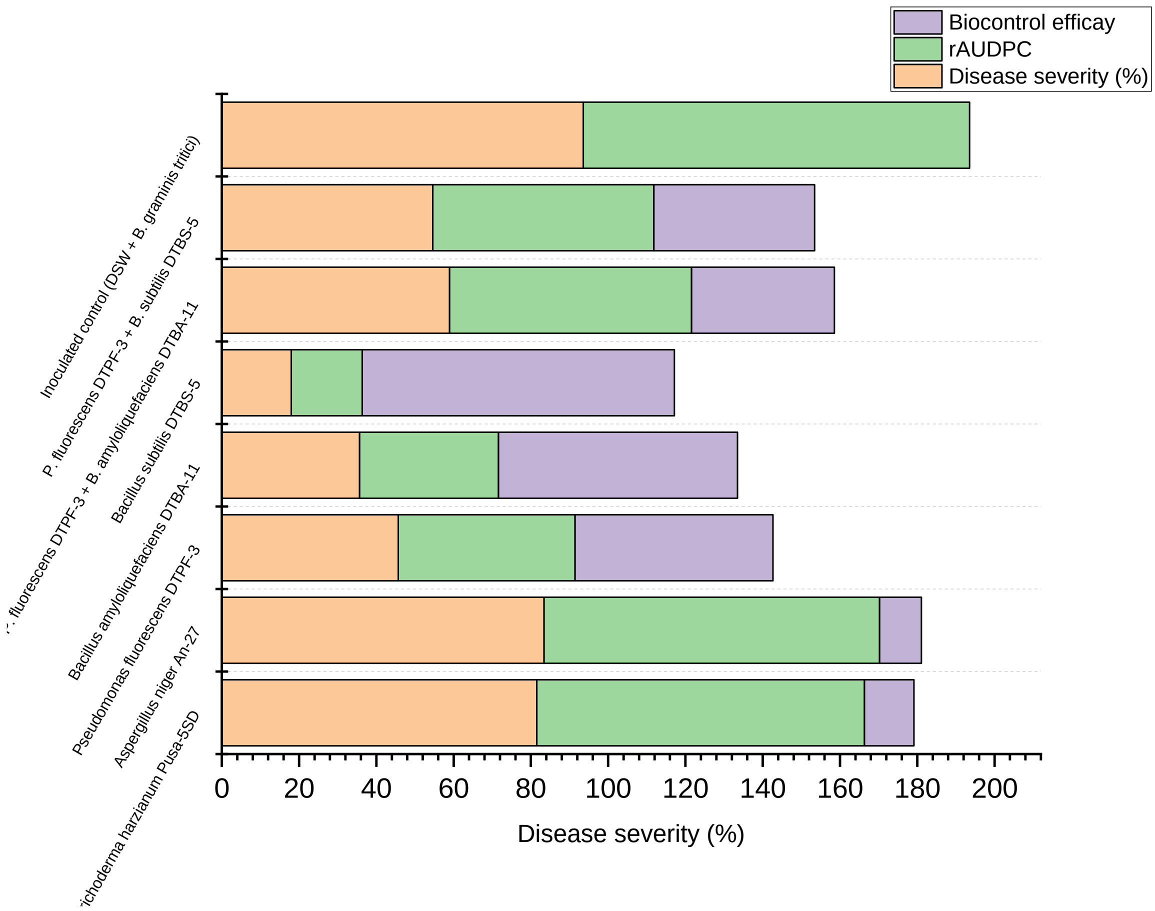
Biocontrol efficay
rAUDPC
Disease severity (%)
Inoculated control (DSW + B. graminis tritici)
P. fluorescens DTPF-3 + B. subtilis DTBS-5
fluorescens DTPF-3 + B. amyloliquefaciens DTBA-11
Bacillus subtilis DTBS-5
Bacillus amyloliquefaciens DTBA-11
Pseudomonas fluorescens DTPF-3
Aspergillus niger An-27
ichoderma harzianum Pusa-5SD
0
20
40
60
80
100
120
140
160
180
200
Disease severity (%)
